# Differentiating Patients at the Memory Clinic With Simple Reaction Time Variables: A Predictive Modeling Approach Using Support Vector Machines and Bayesian Optimization

**DOI:** 10.3389/fnagi.2018.00144

**Published:** 2018-05-22

**Authors:** John Wallert, Eric Westman, Johnny Ulinder, Mathilde Annerstedt, Beata Terzis, Urban Ekman

**Affiliations:** ^1^Department of Public Health and Caring Sciences, Uppsala University, Uppsala, Sweden; ^2^Department of Women's and Children's Health, Uppsala University, Uppsala, Sweden; ^3^Department of Neurobiology, Care Sciences and Society, Karolinska Institutet, Stockholm, Sweden; ^4^Norrlandskliniken, Umeå, Sweden; ^5^Department of Clinical Neuroscience, Karolinska Institutet, Stockholm, Sweden; ^6^Capio Geriatric Hospital, Stockholm, Sweden; ^7^Stockholms Sjukhem, Stockholm, Sweden

**Keywords:** cognitive impairment, dementia, elderly patients, simple reaction time variables, supervised machine learning

## Abstract

**Background:** Mild Cognitive Impairment (MCI) and dementia differ in important ways yet share a future of increased prevalence. Separating these conditions from each other, and from Subjective Cognitive Impairment (SCI), is important for clinical prognoses and treatment, socio-legal interventions, and family adjustments. With costly clinical investigations and an aging population comes a need for more cost-efficient differential diagnostics.

**Methods:** Using supervised machine learning, we investigated nine variables extracted from simple reaction time (SRT) data with respect to their single and conjoined ability to discriminate both MCI/dementia, and SCI/MCI/dementia, compared to—and together with—established psychometric tests. One-hundred-twenty elderly patients (age range = 65–95 years) were recruited when referred to full neuropsychological assessment at a specialized memory clinic in urban Sweden. A freely available SRT task served as index test and was administered and scored objectively by a computer before diagnosis of SCI (*n* = 17), MCI (*n* = 53), or dementia (*n* = 50). As reference standard, diagnosis was decided through the multidisciplinary memory clinic investigation. Bonferroni-Holm corrected *P*-values for constructed models against the null model are provided.

**Results:** Algorithmic feature selection for the two final multivariable models was performed through recursive feature elimination with 3 × 10-fold cross-validation resampling. For both models, this procedure selected seven predictors of which five were SRT variables. When used as input for a soft-margin, radial-basis support vector machine model tuned via Bayesian optimization, the leave-one-out cross-validated accuracy of the final model for MCI/dementia classification was good (Accuracy = 0.806 [0.716, INS [0].877], *P* < 0.001) and the final model for SCI/MCI/dementia classification held some merit (Accuracy = 0.650 [0.558, 0.735], *P* < 0.001). These two models are implemented in a freely available application for research and educatory use.

**Conclusions:** Simple reaction time variables hold some potential in conjunction with established psychometric tests for differentiating MCI/dementia, and SCI/MCI/dementia in these difficult-to-differentiate memory clinic patients. While external validation is needed, their implementation within diagnostic support systems is promising.

## Introduction

Whether abnormal cognitive decline in an elderly individual is diagnosed as mild cognitive impairment (MCI) or dementia, has profound impact on the clinical prognosis, socio-legal interventions, patient self-image, and family adjustments (Ernst and Hay, 1994; Boustani et al., 2003; Ferri et al., 2005; Wimo et al., 2013). The proportion of the population that have dementia is predicted to increase, which apart from the individual suffering also increases the burden on society (Ferri et al., 2005; Wimo et al., 2013). Dementia is characterized by major cognitive decline and problems with activities of daily living (ICD-10)^1^ In contrast, MCI entails minor cognitive decline (Petersen et al., 2001), preserved autonomy in daily functioning (Gauthier et al., 2006), and considerable heterogeneity with diverse clinical manifestations and diverging trajectories regarding somatic and cognitive profiles (Ritchie et al., 2001; Winblad et al., 2004). Although associated with increased risk for developing dementia (Winblad et al., 2004), many diagnosed with MCI remain stable over several years and some even recover (Gauthier et al., 2006). Given the expected growth of these diagnostic groups and the costly diagnostic procedure, more efficient tools for differentiating between MCI and dementia are needed (Boustani et al., 2003).

Diagnostic differentiation of individuals with normal aging, MCI, and dementia is an essential decision made by a primary care physician or, in the more difficult cases, by a multidisciplinary team at a secondary care center (memory clinic). At the memory clinic, a geriatrician usually decides what additional examinations are needed in addition to those already performed in primary care. The most difficult-to-differentiate cases are remitted to a neuropsychologist (Lezak et al., 2012) who regularly performs full neuropsychological assessment (Woodward and Woodward, 2009). A number of these patients show subjective signs of memory impairment but do not fulfill the diagnostic criteria for MCI or dementia. These patients are accordingly labeled with Subjective Cognitive Impairment (SCI). Patients with SCI perform normally in the memory clinical investigation and on psychometric tests, yet they have a heightened risk for developing dementia (Jessen et al., 2014). Full neuropsychological assessment comes with major costs, and there is a need for cost-efficient diagnostic instruments that differentiate in the cognitive borderland between SCI, MCI, and dementia.

The speed and consistency by which we humans process information seem to hold underused diagnostic potential for these patients. The simplest and most widely studied quantification of these cognitive functions is reaction time (Donders, 1969). Reaction time performance reflects basic, bottom-up information processing efficiency, is associated with higher-order cognitive functions (Jensen, 1998; Woodley et al., 2013), and constitutes a proxy for general mental ability (psychometric intelligence) (Jensen, 2006). Simple reaction time (SRT) is the most basic reaction time task, as it only involves one type of response to one type of stimulus. SRT has been described as a relatively pure measure of attention and psychomotor speed, compared to more complex reaction time tasks which in addition also tap inhibitory control and other executive functions (Jensen, 1998). Reaction time variables of central tendency (such as mean, median) have been found to either significantly differ, or to successfully differentiate, between patients with MCI and healthy controls (Dixon et al., 2007; Gorus et al., 2008; Cherbuin et al., 2010; Fernaeus et al., 2013), Alzheimer's disease (AD) and controls (Baddeley et al., 2001; Gorus et al., 2008; Frittelli et al., 2009; Bailon et al., 2010), AD and MCI (Gorus et al., 2008; Frittelli et al., 2009; van Deursen et al., 2009), and non-AD dementia vs. controls (Bailon et al., 2010). More severe neuropathology is consistently indicated by slower and more variable reaction time performance. Compared to reaction time central tendency, reaction time dispersion (variability) has been put forth as particularly sensitive for neural integrity (Hultsch et al., 2002; MacDonald et al., 2008), found to have a dose-response relationship with central nervous system functioning (Burton et al., 2006), and for differentiating amnesic-MCI and AD from healthy controls (Burton et al., 2006; Gorus et al., 2008). Reaction time variability also seems particularly sensitive to cognitive decline (Dixon et al., 2007; Gorus et al., 2008), and for separating MCI from dementia (Tales et al., 2012). Given this previous research, decline in the more basic bottom-up cognitive processes tapped by SRT may provide predictive power for diagnostic accuracy regarding these patients in addition to established psychometric tests which are predominantly designed to tap higher-order cognitive processes such as memory (Lezak et al., 2012).

Aside from the more established measures of reaction time central tendency and dispersion, additional variables extracted from reaction time data (in *italics* below) might prove useful for differentiating SCI, MCI, and dementia. Sustained attention through deliberately *long administrations* with >100 individual reaction time items has been found to differentiate healthy controls from AD (Hellström et al., 1989), and slower reaction time during prolonged administration differentiated MCI from healthy controls (Fernaeus et al., 2013). On the other hand, *shorter administrations* may also be able to separate patient subgroups, for example with only 10 items (Frittelli et al., 2009). Other variables involving specific parts of the marginal density distribution of reaction time responses can be extracted through fitting the Ex-Gaussian function to the marginal reaction time frequency distribution for each individual and calculating the Ex-Gaussian parameters *mu, tau*, and *sigma*. Reaction time data is typically positively skewed (Der and Deary, 2003; van Ravenzwaaij et al., 2011). The majority of fast responses to the left of the reaction time distribution thus fit the Gaussian part (extracted *mu* and *sigma*), and the few slow responses to the right of the reaction time distribution fit the exponential part (*tau*) of the hybrid Ex-Gaussian function (Whelan, 2008) where the latter seem to have specific relevance for attentional lapses (Unsworth et al., 2010). The *worst performances* and *best performances* might also convey relevant information about cognition, seemingly capturing lapses of attention and peak performance, respectively. The Worst Performance Rule hypothesis suggests that worst performances on multi-trial tasks are superiorly indicative of general cognitive ability (Coyle, 2003), and hereby also contradicts classical test theory which instead posits that best performances better capture general cognitive ability (Crocker and Algina, 1986).

The present study sought to evaluate the range of extractable reaction time variables for diagnostic differentiation of SCI, MCI, and dementia. This rendered some specific study design choices worth mentioning in advance. Slower and more variable reaction times are associated with normal aging (Anstey, 1999; Hultsch et al., 2002; Luchies et al., 2002; Deary et al., 2011), lower intelligence (Jensen and Munro, 1979; Deary et al., 2001; Der and Deary, 2003; Woodley et al., 2013), and female sex (Der and Deary, 2006). For diagnostic instruments to be applicable in clinical practice they should be robust against—or control for—such potential confounds. Previous data shows that complex reaction time is more influenced by normal aging (Baddeley et al., 2001; Luchies et al., 2002; Anstey et al., 2005; Der and Deary, 2006; Deary et al., 2011), intelligence (Vernon and Jensen, 1984; Deary et al., 2001), and sex (Der and Deary, 2006), than SRT. Therefore, by parsimony, SRT was favored for the present study. We also employed the Deary-Liewald Reaction Time Task (D-LRTT), a freely available computer program that was validated in 2011 by its constructors (Deary et al., 2011) and has since been used with similarly aged samples (Prado Vega et al., 2013; Vaughan et al., 2014). The D-LRTT constructors claim that it is capable of measuring reaction time accurately with general purpose computers (Deary et al., 2011). The D-LRTT therefore seems ideal for cost-efficient diagnostics and broad clinical applicability. To the extent of our knowledge, this is also the first use of the D-LRTT for differentiating SCI, MCI, and dementia. See the Methods section for further details on the D-LRTT.

Based on the above summary of previous research on reaction time and age-related cognitive decline (Crocker and Algina, 1986; Hellström et al., 1989; Jensen, 1998, 2006; Baddeley et al., 2001; Hultsch et al., 2002; Coyle, 2003; Der and Deary, 2003; Burton et al., 2006; Dixon et al., 2007; Gorus et al., 2008; MacDonald et al., 2008; Whelan, 2008; Frittelli et al., 2009; van Deursen et al., 2009; Bailon et al., 2010; Cherbuin et al., 2010; Unsworth et al., 2010; van Ravenzwaaij et al., 2011; Tales et al., 2012; Fernaeus et al., 2013; Woodley et al., 2013), we hypothesized that (1) SRT variables would display the overall pattern of dementia > MCI > SCI, (2) that SRT variables would differentiate diagnostic groups comparably to clinical variables when used as predictors by themselves and conjointly with established psychometric tests commonly used in neuropsychological assessment, and (3) compared to single-predictor modeling, multivariate modeling would result in improved differential diagnostic accuracy.

## Methods

### Prospective design

The present study design conforms to the The Standards for Reporting of Diagnostic Accuracy when evaluating novel diagnostic instruments (Bossuyt et al., 2003). SRT data was anonymized before being gathered, through the patient receiving a code directly in the D-LRTT software from the clinician that started the test. The D-LRTT result was thereafter automatically registered by the computer. Other neuropsychological tests were then administered, and diagnosis was thereafter decided. The assessing neuropsychologist administered and scored the established neuropsychological tests as part of the reference standard (Memory clinic investigation), except for the Mini Mental State Examination (MMSE) (Folstein et al., 1975; Palmqvist, 2011).

### Patients and clinical setting

Data was collected from 23rd October 2015 to 7th of October 2016 at the Bromma geriatric hospital's memory clinic in the western part of Stockholm, Sweden. Consecutive patients remitted to neuropsychological assessment with “subjective cognitive disorder” (International Statistical Classification of Diseases and Related Health Problems-Tenth Revision [ICD-10: R41.8A])^1^ were eligible for inclusion. One patient aborted the reaction time task, seven had incomplete reaction time data, one had inconclusive diagnosis, and were excluded. The final sample consisted of 120 patients (17 SCI, 53 MCI, and 50 dementia).

### Index test (D-LRTT)

The D-LRTT presents each SRT item as a black “X” visible inside a white box in the center of an otherwise darkblue computer screen. The screen was displayed at armslength from the sitting patient. Patients responded to items by quickly pressing the keyboard Space bar with the index finger of their preferred hand and releasing it swiftly. The D-LRTT registered 107 correct item responses corresponding to approximately 5 min of testing (estimated before study start). Item error presets were ≥150 ms for the lower and ≤ 1,500 ms for the upper bound. Answers within these bounds were considered correct. Inter-item delay was randomized within 1,000–3,000 ms. Instructions focused on vigilance and speed. The single fastest and slowest responses were deleted to limit outlier influence (e.g., Coyle, 2003). This rendered a 105 item run (5-minute condition) from which we also extracted an abbreviated run including the first 21 items (1-minute condition) for each patient. The three Ex-Gaussian variables mu (μ), sigma (σ), and tau (τ) were extracted from the distribution of the 105 responses. Respectively, μ and σ signifies the mean and standard deviation of the Gaussian component (the peak and spread of the “hill” to the left in a reaction time distribution), while τ is the mean of the exponential component (the “long tail” to the right of the same distribution) of the Ex-Gaussian function. The Worst Performance rule variables were constructed through ordering the 105 responses from worst to best, creating quintile bins with 21 items per bin, and calculating the median of the worst (WP1) and best (WP5) performance bins. Together with the arithmetic mean and dispersion variables extracted from both the first minute of testing (SRTS) and the full 5 min testing (SRT), this rendered a total of nine different SRT variables evaluated for diagnostic accuracy. See Table 1 for details.

**Table 1:** Description of the nine Simple Reaction Time (SRT) predictor variables gathered from each patient and used for diagnostic differentiation.

**Variable abbreviation**	**Description**
SRTS-mean	Mean of the first 21 item responses
SRTS-sd	Standard deviation of the first 21 item responses
SRT-mean	Mean of the 105 item responses
SRT-sd	Standard deviation of the 105 item responses
SRT-μ	Mean of the Ex-Gaussian fast responses
SRT-σ	Standard deviation of the Ex-Gaussian fast responses
SRT-τ	Mean of the Ex-Gaussian slow responses
SRT-WP1	Median of the 21 fastest item responses
SRT-WP5	Median of the 21 slowest item responses

*Mean is the arithmetic mean. Error responses defined as either ≤ 150 or ≥1,500 ms were automatically excluded by the D-LRTT software*.

### Reference standard (memory clinic investigation)

All patients were referred from a primary care facility after their basal dementia examination, which included anamnesis, evaluation of physical and psychological status, blood sampling, a Computer Tomography (CT) scan, a clock test, and the MMSE. The memory clinic investigation that followed is an accepted reference standard for clinical classification of SCI, MCI, and dementia (Appels and Scherder, 2010). In Sweden, it consists of standardized examinations over several days performed by a multiprofessional team of clinicians. The investigation began with the geriatric examination including anamnesis and neurological/affective/cognitive screening. Thereafter, additional interventions were decided by the geriatrician (Activities of Daily Living evaluation, Neuropsychological assessment, lumbar puncture (LP), structural (CT or MRI), or functional (EEG) neuroimaging. A MMSE score of ≥24 was normally required for neuropsychologist referral. All included patients performed the neuropsychological assessment. This involved an approximately 120 min long, tailored battery of validated neuropsychological tests for assessing cognitive dysfunction typically associated with elderly cognitive decline. There was a brief, scheduled pause halfway through the assessment. The reference standard test battery included five subtests from the 4th version of the Wechsler Adult Intelligence Scale (WAIS-IV): Arithmetic (AR), Digit Span (DS), Information (IN), Block Design (BD), Similarities (SI), two subtests from the Delis-Kaplan Executive Function System (D-K EFS): Trail Making Test (TMT), Verbal Fluency (VF). The battery also included Logical Memory (LM) from the 3rd version of the Wechsler Memory Scale (WMS-III), Rey Auditory Verbal Learning Test (RAVLT), Rey-Osterrieth Complex Figure Test (RCFT), Boston Naming Test-60 item version (BNT-60), Luria Clocks, and Figure Copying. At the subsequent diagnostic conference, the clinical team decided formal diagnosis according to the ICD-10. The Petersen's diagnostic criteria (Petersen, 2004) were used in conjunction with ICD-10: F06.7 for MCI, and corresponding ICD-10 subtypes for dementia^1^.

### Select parts of the reference standard

Since cost-efficiency is a crucial goal of the present study, only two variables from the neuropsychological assessment standard were selected for predictive evaluation (DS total score and RAVLT total learning). These two established test variables influenced the final diagnosis yet were selected so that this influence was only minor (>100 variables from >20 psychometric tests are used for final diagnosis). With that said, DS and RAVLT had a slight advantage to the index SRT test result since the latter was neither allowed diagnostic influence nor available for clinicians to survey before formal diagnosis. Unadjusted raw scores were used for all psychometric tests, except for the premorbid full-scale intelligence estimate (IN FSIQ). IN FSIQ is commonly used as a proxy for premorbid IQ because the cognitive functions it measures (semantic, aquired knowledge, and verbal reasoning) are considered robust to early cognitive impairment (Lezak et al., 2012). Accordingly, the raw score on IN was not used. The IN raw scores were instead translated to the age-adjusted WAIS-IV norm and are reported herein as T-scores with mean = 50 and sd = 10.

### The predictor set

The full 11 predictor set included the 9 SRT variables, DS, and RAVLT.

### Basic statistics

We report descriptive data by diagnostic group and total sample in mean (sd) or count (%). Since the purpose of the paper was pure prediction, we kept data as raw as possible, rather than applying transformations seeking to normalize that which is inherently non-normal. Consequently, we also used mostly non-parametric inferential tests. We applied the Kruskal-Wallis rank sum test, and the Pearson Chi-square test (or Fisher's exact test with its Fortran extension as appropriate) for continuous and categorical variables, respectively. If significant, these were respectively followed up with either post-hoc Dunn tests, or Chi-square/Fisher tests for each pair of diagnostic groups. *P*-values from post-hoc testing are Bonferroni-Holm corrected due to multiple comparisons (Holm, 1979). We set statistical significance to 5%, and report 95% confidence intervals with point estimates.

### Predictive modeling

Diagnostic accuracy of the 11 variables (now treated as predictors) was investigated with a supervised machine learning approach (Jordan and Mitchell, 2015). Pseudorandomization with a constant starting seed was used throughout to ensure reproducibility. Because there were relatively few cases compared to variables, Support Vector Machines (SVM) were chosen (Boser et al., 1992; Cortes and Vapnik, 1995). SVMs are a flexible group of models that typically perform well on classification problems with relatively few samples (patients) compared to the number of dimensions (variables). Specifically, we employed a soft margin SVM with a non-linear (radial basis) kernel (Boser et al., 1992; Cortes and Vapnik, 1995). A soft margin SVM allows for spatial overlap between classes across the separator hyperplane when fitted to data using the few closest datapoints with opposite class labels (support vectors) so that the margin between them is maximized. For tuning the hyperparameters of the SVM, we first initiated 10 instances of random hyperparameter settings as initial training. For each of these training iterations, the Cost-function (degree of spatial overlap between classes) was allowed to vary between log −5 and log 15, and Sigma (the radial basis kernel hyperparameter) to vary between log −10 and log 5. Thereafter, we fed these hyperparameter settings with their corresponding performance to the Bayesian optimization procedure, and tuning of the hyperparameters was continued (Bilj et al., 2016; Shahriari et al., 2016). Bayesian optimization ran for 40 additional training iterations with the upper bound of the Gaussian process as acquisition function. As Gaussian process kernel we used the squared exponential (Yan, 2016).

During feature selection and model estimation we applied cross-validation (CV), albeit two variants of it separately. To ensure that we did not overfit during feature selection prior to building the final multivariable models, we applied resampled recursive feature elimination optimized on classification accuracy. Recursive feature elimination is an iterative greedy algorithm which prunes away the lowest ranking features one at a time until the optimal set of features are found (Kohavi and John, 1997; Guyon et al., 2002). This recursive feature elimination was resampled with three stochastic repeats of 10-fold CV (3 × 10-fold CV). The basis of repeated n × 10 CV is the same as one pass of regular 10-fold CV but then repeated n additional times on the same data with all observations randomly assigned to the 10-folds per each pass. The result is then averaged just as for one pass of regular CV.

For hyperparameter tuning and parameter estimation of the two final multivariable models, we again optimized on classification accuracy but instead applied leave-one-out CV. The best performing tuning setting was then used for the final model which was fitted to the whole dataset and model performance was calculated. Although we would have preferred to use 10-fold CV throughout with additional model validation with unseen (hold-out) data (Wallert et al., 2017b), the moderate sample size suggested leave-one-out CV as a useable option (Hastie et al., 2009; Månsson et al., 2015). The bias-variance tradeoff also suggested leave-one-out CV over 10-fold CV insofar that the former has lower bias than the latter. For the hyperparameter tuning part, there remained a chance for overfitting (see the section Discussion regarding the need for external model validation).

For both classification problems (MCI/dementia; SCI/MCI/dementia), we estimated (a) crude models for each of the 11 predictors separately, and (b) the final two multivariable models which only included the predictors selected by recursive feature elimination. Accuracy with 95% CIs was used as the main performance metric throughout. For each of the two final models, we also report the sensitivity, specificity, positive/negative predictive value, and the confusion matrices result. We examine the pretest probabilities and posttest probabilities for these two final models and exemplify the clinical use of the three-class model with a hypothetical new patient. Finally, we implement the final models in an online decision support system (see the Results section for details).

### Software

Data was prepared in Excel 2010 (Microsoft Corp, Washington), and analyzed in R version 3.3.2 (R Development Core Team, Vienna) (R Development Core Team, 2015) using packages *base, dunn.test, fifer, plyr, pROC, psych, retimes*, and *stats*. Training and testing of the machine learning models specifically employed the packages *caret* (Kuhn, 2008)*, kernlab* (Karatzoglou et al., 2004), and *rBayesianOptimization* (Yan, 2016).

## Results

Dementia subdiagnoses in order of frequency were: AD (17/50 cases, 34%), mixed AD and vascular (15/50, 30%), vascular (7/50, 14%), other specified (Lewy Body, Parkinson's) (6/50, 12%), and unspecified (5/50, 10%). The patient age range was 65–95 years.

Demographic and clinical data are presented in Table 2. For group comparisons, age did not differ significantly between diagnostic groups (rank sum χ^2^ = 1.568, *P* = 0.457) but education did (χ^2^ = 11.781, *P* = 0.003), with SCI patients being more educated than MCI (Dunn test = 2.830, *P* = 0.005), and dementia (3.408, *P* = 0.001) patients. Sex proportional differences were significant (Pearson χ^2^ = 7.727, *P* = 0.021) showing more males in the MCI group. Group proportions of possible depression (extended Fisher's exact test *P* = 1) and patient follow-up (*P* = 0.720) did not differ significantly. Sex proportions were balanced in the total sample.

**Table 2:** Demographics, clinical characteristics, and interventions by diagnostic group and total sample.

	**SCI (*N* = 17)**	**MCI (*N* = 53)**	**Dementia (*N* = 50)**	**Total (*N* = 120)**
**DEMOGRAPHICS**				
Age (years)	74.8 (5.0)	77.2 (7.9)	77.3 (6.8)	76.9 (7.1)
Education (years)	15.6 (3.9)	12.4 (4.1)	11.6 (3.6)	12.5 (4.1)
Men	12 (71)	30 (57)	18 (36)	60 (50)
Women	5 (29)	23 (43)	32 (64)	60 (50)
**COMORBID CONDITIONS**				
Possible depression	1 (6)	6 (11)	5 (10)	12 (10)
**CLINICAL EXAMINATION**				
MRI	8 (47)	21 (40)	22 (44)	51 (43)
EEG	0 (0)	3 (7)	8 (16)	11 (9)
LP	8 (47)	29 (55)	33 (66)	70 (58)
Geriatrician	17 (100)	53 (100)	50 (100)	120 (100)
Anamnesis	17 (100)	53 (100)	50 (100)	120 (100)
Work therapist	2 (12)	12 (23)	26 (52)	40 (33)
Neuropsychologist	17 (100)	53 (100)	50 (100)	120 (100)
Follow-up	2 (12)	11 (21)	8 (16)	21 (18)

*Data are decimal mean (sd) or integer count (%). Possible depression was assessed by the clinical psychologist. MCI, Mild Cognitive Impairment; SCI, Subjective Cognitive Impairment; MRI, Magnetic Resonance Imaging; EEG, Electroencephalography; LP, Lumbar Puncture*.

Groups were then compared on each psychometric test. As evident in Table 3, psychometric performance scores were consistently best for SCI, worst for dementia, and intermediate for MCI. Group differences were generally larger between SCI and MCI, than between MCI and dementia. Because inferential group comparisons on these variables were ancillary in this differential diagnostic paper, these results are reported in the Appendix.

**Table 3:** Psychometric performance by diagnostic group and total sample.

	**SCI (*N* = 17)**	**MCI (*N* = 53)**	**dementia (*N* = 50)**	**Total (*N* = 120)**
SRTS-mean	330.9 (30.9)	386.0 (105.1)	414.8 (120.9)	390.2 (108.3)
SRTS-sd	47.1 (12.0)	100.9 (76.4)	101.2 (54.9)	93.4 (64.5)
SRTS-median	323.4 (36.1)	359.6 (102.0)	390.4 (114.2)	367.3 (103.0)
SRT-mean	337.0 (33.9)	381.3 (80.1)	427.1 (123.1)	394.1 (101.0)
SRT-sd	54.1 (14.5)	101.6 (54.7)	114.9 (58.8)	100.4 (56.2)
SRT-median	325.8 (34.6)	356.9 (78.7)	403.6 (118.4)	372.0 (97.3)
SRT-μ	288.9 (35.2)	280.8 (78.3)	324.0 (113.3)	300.0 (92.5)
SRT-σ	29.7 (14.7)	51.4 (39.7)	58.5 (45.3)	51.3 (40.6)
SRT-τ	48.1 (20.6)	100.5 (59.4)	103.1 (55.0)	94.2 (56.6)
SRT-WP1	400.6 (43.1)	494.7 (141.6)	566.8 (193.9)	511.4 (166.3)
SRT-WP5	284.8 (24.3)	292.1 (39.9)	317.7 (73.1)	301.7 (56.3)
IN FSIQ (*N* = 119)	60.1 (7.3)	51.9 (8.5)	45.0 (9.7)	50.3 (10.2)
MMSE-SR (*N* = 117)	28.5 (1.1)	27.1 (2.1)	25.3 (2.4)	26.5 (2.4)
BD (*N* = 119)	36.2 (9.0)	26.9 (7.3)	22.8 (7.2)	26.6 (8.7)
DS	24.9 (5.0)	22.1 (3.8)	19.1 (4.2)	21.2 (4.6)
BNT-60 (*N* = 113)	55.1 (3.5)	49.8 (9.5)	46.8 (9.5)	49.3 (9.3)
RAVLT	45.6 (9.0)	33.2 (9.8)	27.9 (8.5)	32.8 (10.8)
LM (*N* = 108)	12.7 (4.6)	7.4 (4.6)	4.3 (3.7)	6.9 (5.1)
RCFT (*N* = 97)	17.5 (5.4)	9.7 (5.8)	4.7 (4.7)	9.4 (6.8)
TMT4 (*N* = 110)	107.0 (35.6)	162 (54.7)	220.0 (40.9)	176.7 (61.2)
VFT-shifting (*N* = 119)	12.4 (2.2)	9.9 (2.7)	7.2 (3.2)	9.2 (3.4)
Clock test (*N* = 118)	4.2 (1.1)	3.0 (1.2)	1.6 (1.2)	2.6 (1.5)
Draw Cube (*N* = 118)	0.9 (0.2)	0.7 (0.4)	0.3 (0.4)	0.6 (0.4)
Draw Cross (*N* = 118)	0.8 (0.4)	0.6 (0.4)	0.3 (0.4)	0.5 (0.5)

*Values are mean (sd). Reaction time variables are in milliseconds. Raw scores are presented except for the premorbid intelligence estimate (IN) which is age adjusted and rescaled to the WAIS-IV norm T-scores with mean = 50 and sd = 10. MCI, Mild Cognitive Impairment; SCI, Subjective Cognitive Impairment; BD, Block Design; BNT-60, Boston Naming Test−60 item version; DS, Digit Span—Total score; IN, WAIS-IV Information; LM, Wechsler Memory Scale III/Logical Memory—Total Delayed Recall; MMSE-SR, Mini Mental State Examination—Swedish Revision; RAVLT, Rey Auditory Verbal Learning Test—Total Learning; RCFT, Rey-Osterrieth Complex Figure Test—Delayed Recall; SRT, 5-min Simple Reaction Time (105 item responses); SRTS, 1-min Simple Reaction Time (21 item responses); TMT4, D-K EFS Trail-Making Test—Shifting; VFT, D-K EFS Verbal Fluency Test; WP1, Worst performances; WP5, Best performances; μ, mean of the Gaussian part of the Ex-Gaussian distribution; σ, sd of the Gaussian part of the Ex-Gaussian distribution; τ, mean of the exponential part of the Ex-Gaussian distribution*.

**Figure 1:**
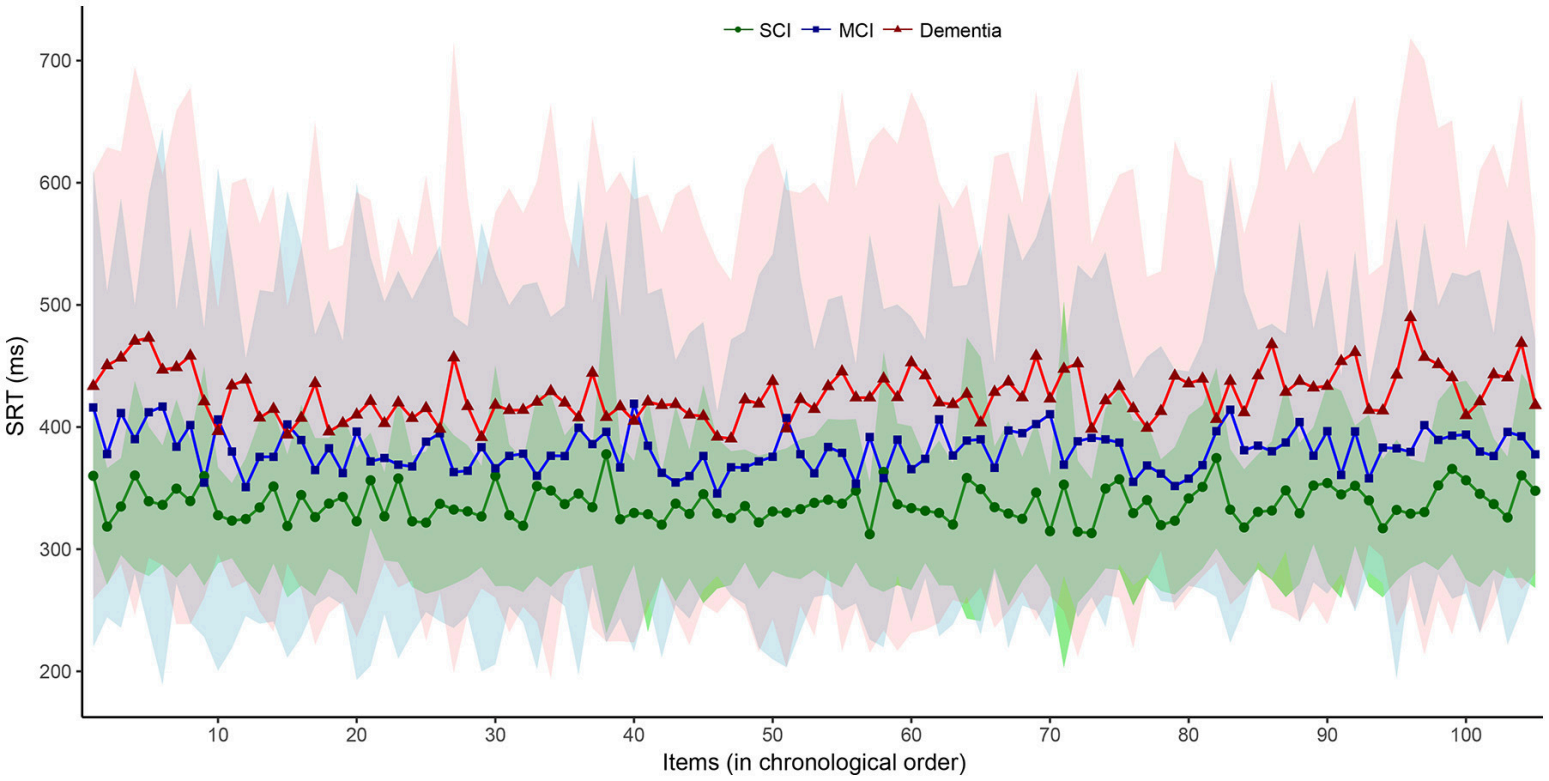
Item-by-item SRT performance in chronological order by diagnostic group. Interconnected dot-shapes represent each group mean for each item response of the 105 items over the 5-min SRT administration from start to finish. Shaded areas represent the corresponding ± one standard deviation from the mean for each item response with the SCI group in green overlaying the MCI group in blue overlaying the dementia group in red. MCI, Mild Cognitive Impairment. SCI, Subjective Cognitive Impairment.

**Figure 2:**
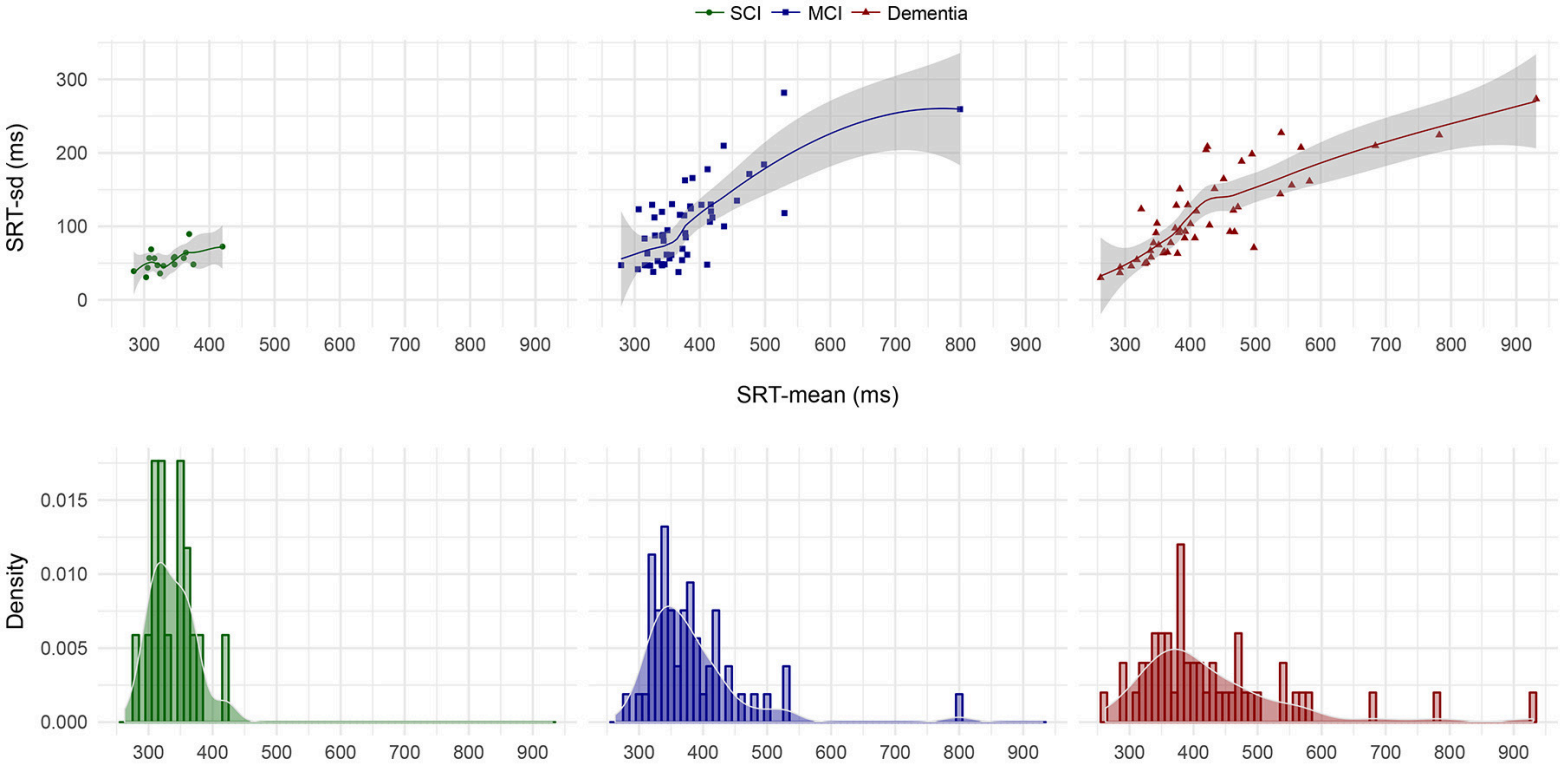
SRT-sd as a function of SRT-mean for each patient by diagnostic group and SRT-mean density by diagnostic group. Points in the upper three facets represent each individual patient's standard deviation as a function of their arithmetic mean on the 105 item SRT testing. The lines and shaded error bars represent fitted loess functions with 95% CIs. Histograms in the lower three facets represent the counts per diagnostic group of patients' mean SRT that fall within 10 ms bins where bar heights indicate relative counts. Overlaid on the histograms are the corresponding density distributions. MCI, Mild Cognitive Impairment; SCI, Subjective Cognitive Impairment.

We then plotted the 105 SRT responses by group in (i) chronological, (ii) central tendency vs. dispersion, and (iii) density form. Visual inspection of the chronological plot in Figure 1 reveal a consistent pattern over time where the SCI group was faster and particularly less variable than the MCI group, which in turn was faster and less variable than the dementia group. In Figure 2, the upper three facets shows each patient's SRT-sd vs. SRT-mean over 105 items by group with fitted non-parametric functions, while the lower three facets again shows each patient's SRT means by group but now as histograms with overlaying density functions. Notice in Figure 2 the increasingly elongated shape of SRT distributions across patient groups from SCI to MCI to dementia.

Next, the different SRT variables and additional variables were evaluated with respect to their differential diagnostic accuracy (details in the Methods section, heading “Predictive modeling”). The single-predictor accuracy of each of the 11 psychometric predictors and three demographic predictors are available in Table 4. For classifying MCI/dementia correctly, the best performing reaction time variables were SRTS-mean, SRT-μ, and SRT-sd in decreasing order of accuracy. SRT-mean and SRT-μ were also comparable to the top-scoring, established neuropsychological tests RAVLT and DS. For the SCI/MCI/dementia classification, SRT-τ, SRT-μ, and SRT-WP1 performed best in the given order. SRT-τ and SRT-μ also showed similar accuracy as DS and RAVLT. For both two-class and three-class classification, SRT worst performances (WP1) performed slightly better on this sample, compared to SRT best performances (WP5). Age and education were not useful as single classifiers. By itself, sex held some classifier merit for the two-class and three-class problem. As single predictors, the DS and RAVLT performed slightly better than the best SRT variables on both classification problems with the exception of SRT-τ having higher accuracy than RAVLT on the three-class problem.

**Table 4:** Diagnostic accuracy of single predictors for differentiating MCI/dementia, and SCI/MCI/dementia.

	**MCI/Dementia**			**SCI/MCI/Dementia**		
	**(*N* = 103)**			**(*N* = 120)**		
**Predictors**	**Acc^a^**	**CI (95 %)**	***P***	**Acc^a^**	**CI (95%)**	***P***
SRTS-mean	0.864	0.783, 0.924	<0.001	0.533	0.440, 0.625	0.135
SRTS-sd	0.670	0.570, 0.759	0.004	0.492	0.399, 0.585	0.624
SRT-mean	0.757	0.663, 0.836	<0.001	0.617	0.524, 0.703	<0.001
SRT-sd	0.796	0.705, 0.869	<0.001	0.425	0.335, 0.519	1
SRT-μ	0.845	0.760, 0.909	<0.001	0.692	0.601, 0.773	<0.001
SRT-σ	0.544	0.443, 0.642	0.623	0.450	0.359, 0.544	1
SRT-τ	0.680	0.580, 0.768	0.002	0.733	0.645, 0.810	<0.001
SRT-WP1	0.767	0.673, 0.845	<0.001	0.650	0.558, 0.735	<0.001
SRT-WP5	0.728	0.632, 0.811	<0.001	0.625	0.532, 0.712	<0.001
Age	0.563	0.462, 0.661	0.563	0.642	0.549, 0.727	<0.001
Education	0.534	0.433, 0.633	0.623	0.458	0.367, 0.552	1
Sex	0.816	0.727, 0.885	<0.001	0.708	0.618, 0.788	<0.001
DS	0.893	0.817, 0.946	<0.001	0.758	0.672, 0.832	<0.001
RAVLT	0.884	0.805, 0.938	<0.001	0.692	0.601, 0.773	<0.001

a *Leave-one-out cross-validation accuracy. Each predictor performance obtained with a soft-margin radial basis function support vector machine tuned over 10 random and 40 Bayesian optimization hyper-parameter settings. P-values are Bonferroni-Holm corrected for each classification problem (column), and signifies one-sided tests of accuracy (single-predictor model > No Information Rate). CI, Confidence Interval; MCI, Mild Cognitive Impairment; SCI, Subjective Cognitive Impairment; DS, WAIS-IV Digit Span—Total Score; RAVLT, Rey Auditory Verbal Learning Test—Total learning; SRT, 5-min Simple Reaction Time (105 item responses); SRTS, 1-min Simple Reaction Time (21 item responses); WP1, Worst performances; WP5, Best Performances; μ, mean of the Gaussian part of the Ex-Gaussian distribution; σ, sd of the Gaussian part of the Ex-Gaussian distribution; τ, mean of the exponential part of the Ex-Gaussian distribution*.

We then ran the resampled recursive feature elimination procedure to extract the optimal feature subset for each of the two final multivariable models. Recursive feature elimination selected seven predictors as optimal for both classification problems. Each subset included the two established tests DS and RAVLT along with five additional SRT variables. We thereafter fitted the two final models with these subsets, first tuning the model hyperparameters with outer CV and then refitting the model with the best tuning setting on the respective whole dataset. The resulting two models constitute the main study result. For differentiating MCI/dementia, the final model solved this two-class problem with good accuracy (Accuracy = 0.806 [0.716, 0.877], *P* < 0.001). For differentiating SCI/MCI/dementia, the final model was somewhat accurate on this three-class problem (Accuracy = 0.650 [0.558, 0.735], *P* < 0.001). We thereafter ran recursive feature elimination with only the SRT variables. Naturally, these models were weaker yet still showed some classification accuracy for differentiating MCI/dementia (Accuracy = 0.680 [0.580, 0.768], *P* < 0.001), and SCI/MCI/dementia (Accuracy = 0.492 [0.399, 0.585], *P* = 0.156). See Table 5 for further details on the two final models, including the actual classification results in confusion matrices with additional performance metrics.

**Table 5:** Performance of the two final multivariable models for diagnosing MCI/dementia and SCI/MCI/dementia, respectively.

MCI/Dementia^*^				SCI/MCI/Dementia^**^		
		**Observed**		**Observed**		
		**(*N* = 103)**		**(*N* = 120)**		
		**MCI**	**Dementia**	**SCI**	**MCI**	**Dementia**
Predicted	SCI	–	–	5	3	0
	MCI	53	20	9	47	24
	Dementia	0	30	3	3	26
Accuracy^a^ (95% CI)		0.806 (0.716, 0.877)		0.650 (0.558, 0.735)		
No information rate		0.515		0.442		
*P*-value		<0.001		<0.001		
Kappa		0.607		0.402		
Sensitivity		1		0.294	0.887	0.520
Specificity		0.600		0.971	0.508	0.914
Pos predictive value		0.726		0.625	0.588	0.813
Neg predictive value		1		0.893	0.850	0.727
Prevalence		0.515		0.142	0.442	0.417

a *Leave-one-out cross-validation accuracy*.

* *Includes DS, RAVLT, SRT-WP1, SRT-sd, SRT-μ, SRT-mean, and SRTS-sd*.

** *Includes DS, RAVLT, SRT-WP1, SRT-sd, SRTS-sd, SRT-τ, and SRT-mean*.

*Correct predictions in each confusion matrix are diagonal from upper left to lower right. Input predictors are selected through 3 × 10-fold cross-validated recursive feature elimination. Performance metrics are calculated once with MCI as target class for the two-class model. This calculation is repeated for each diagnosis versus remaining diagnoses (one versus all) for the three-class model. Bonferroni-Holm corrected P-values are from a one-sided binomial test of accuracy for each single-predictor model > the corresponding No Information Rate (largest percentage class). DS, Digit Span—Total score; RAVLT, Rey Auditory Verbal Learning Test—Total learning; MCI, Mild Cognitive Impairment; SCI, Subjective Cognitive Impairment*.

Here, is an exemplification of running the three-class SCI/MCI/dementia model from Table 5 on a new patient at the memory clinic actualized for neuropsychological assessment. Before assessment, there is an average 14.2, 44.2, and 41.7% base rate probability that the patient will later receive a SCI, MCI, and dementia diagnosis, respectively (pre-test probability). Now imagine that the patient performs the selected psychometric tests and the result is fed into the model. The model will then output a prediction, and if the model suggests that the patient has SCI, there is now a 62.5% chance that the patient will later receive a SCI diagnosis after completing the full memory clinic examination (positive predictive value). If the model instead suggests either MCI or dementia for the patient, there is only a 10.7% chance that the patient will later receive a SCI diagnosis after completed examination. If the model instead predicts MCI, the patient has a 58.8% chance to later receive MCI diagnosis. If the model does not predict MCI, the chance for later MCI diagnosis drops to 15.0%. Finally, if the model predicts dementia, the patient has a 72.7% chance of later dementia diagnosis, yet if the model does not suggest dementia, the risk for final dementia diagnosis drops to 27.3%.

### Implementation

For research and exploratory purposes, we also implement the two final models (Table 5) in a decision support application, made available for free with the present publication at http://wallert.se/mcdpi^2^. The Memory Clinic Diagnostic Prediction Instrument (MC-DPI) supports both mobile and stationary platforms. After calculating the predictor values for a new patient, one simply inputs these values in their designated fields and clicks the “Calculate” button. This returns the predicted diagnosis for the patient. The application is built with R and Ruby on Rails and hosted on a cloud-based server (Ubuntu 14.04). The input values are fed to an R backend which runs the values through the chosen prediction model and returns the predicted diagnosis. Although the present models are cross-validated, they are in need of external validation with a larger sample of unseen data. We take no responsibility for the use or misuse of these models.

## Discussion

In a difficult-to-differentiate memory clinic sample referred to full neuropsychological assessment we showed that SRT variables hold potential for differentiating MCI/dementia, and SCI/MCI/dementia. SRT variables displayed the overall pattern of dementia > MCI > SCI (hypothesis 1). SRT variables held some classifier merit by themselves but the multivariable machine learning procedure showed that the best solution to both diagnostic problems included SRT variables in conjunction with established psychometric tests (hypotheses 2 and 3).

Interestingly, the mean of the slow reaction time responses (Ex-Gaussian τ) performed reasonably on the more difficult task of differentiating SCI/MCI/dementia. This might relate to other findings suggesting that τ captures attentional lapses (e.g., Unsworth et al., 2010). The slowest responses on SRT tasks are also the “worst” responses and are directly related to the Worst Performance Rule paradigm which suggests worst performances as a better measure of general cognitive ability than best performances (Coyle, 2003). The present study found a higher accuracy for worst performances than best performances, although the difference was small and might be stochastic. The Worst Performance Rule variables have not been previously applied to differential diagnostics of these clinical groups and they might constitute a new psychometric route to improved understanding and prediction of these patients' deteriorating cognition. To some extent, the present results also add to the bulk of existing evidence suggesting that variability-of-processing is more sensitive to neurodegenerative cognitive decline in elderly than speed-of-processing (e.g., Gorus et al., 2008; Tales et al., 2012; Wallert et al., 2017a).

We investigated SRT relative to other established cognitive tests which items have been developed over decades, and are known to tap much more complex cognitive abilities such as memory. It is intuitively quite surprising that such a simple task as SRT can perform reasonably well-compared to other established psychometric tests in terms of both binary and trinary classification of complex cognitive decline and pathology. Importantly, this result was obtained with the most difficult to diagnose patients in the borderland of SCI/MCI/dementia, for whom diagnostics demanded full neuropsychological assessment. The bottom-up strength of SRT herein underscores how more complex cognitive operations are dependent on such basic mental operations as those captured by SRT (Baddeley et al., 2001; Hultsch et al., 2002; Burton et al., 2006; Jensen, 2006; Dixon et al., 2007; Gorus et al., 2008; MacDonald et al., 2008; Frittelli et al., 2009; Bailon et al., 2010; Cherbuin et al., 2010; van Ravenzwaaij et al., 2011; Tales et al., 2012). The combination of bottom-up SRT variables and established top-down psychometric tests seems particularly potent as they together yielded the best diagnostic performance for both classification problems in the present study.

Practically, SRT holds many clinical benefits compared to the established, more complex tests. Many established tests are verbal and hence biased if the patient does not speak the native language, have low education or difficulties with verbal communication (Jensen, 2006). In this regard, SRT tasks are highly culture-fair and skill-fair. Another benefit is the level of data obtained. Reaction time tasks gather data at a high level of scale. Most psychological tests do not gather data at this level; a few promise interval data yet most deliver ordinal data. The unbiased and precise computer scoring of reaction time is also a substantial benefit compared to most other neuropsychological tests which are inherently biased to some extent by the human administering them. In this often neglected regard, reaction time tests possess superior reliability and validity compared to most other psychometric tests. Reaction time items are also answered quickly, compared to items from more complex tests. Reaction time tasks therefore generate superior reliability per time unit compared to other tests which items take longer time to answer.

There are important limitations to the present study. A larger sample would have allowed for external model validation, including investigation of age, gender, intelligence, and diagnostic subtypes, and statistical inference regarding the differences in diagnostic accuracy between SRT variables and established tests found in the present study. These areas constitute important areas for future differential diagnostic research, which in turn depends on the search for new cost-effective diagnostic predictors, and the collection of more high-quality data. There is also the need for adapting diagnostic models to the clinical situation which depends on more than just the model accuracy. During feature selection, we consider overfitting as unlikely since we applied an algorithmic selection procedure that was robustly resampled with 3 × 10-fold CV. For both classification problems, this recursive feature elimination procedure selected highly similar features as the single-predictor accuracy ranking of predictors did. For the tuning of model hyperparameters, we applied Bayesian optimization within leave-one-out CV resampling. Hence, we prioritized low bias at the cost of potentially higher variance by fitting as much of the data as possible yet still cross-validating the results. This might have induced some overfitting at this step, i.e., the risk of these models not generalizing to new cases and future external validation is again suggested. Unfortunately, having enough data to allow for the optimally robust control for overfitting is rarely the case—especially so in research using representative, high-quality clinical data from specialized healthcare with gold standard diagnostics. Although most confounding variables were documented, the modest sample size did neither allow for statistical control for confounding nor causal inference. This was of course a deliberate limitation because our main aim was an ecologically and clinically valid study focused on the predictive diagnostic needs of the specific clinical context. As external validity is a prerequisite for clinical applicability (Rothwell, 2005), specifically due to both frequent comorbidity (Fried et al., 2004) and the etiological heterogeneity underlying dysfunctional cognition in elderly patients (Woodward and Woodward, 2009; Ramakers and Verhey, 2011), new instruments should be evaluated with ecologically valid samples (Greenhalgh, 1997). Regarding causality, we refer to the impressive amount of previous reaction time research (e.g., Jensen, 2006). Another limitation was that the established psychometric tests, but not SRT, were to a limited degree allowed to influence final diagnosis. SRT variables were therefore somewhat handicapped in comparison. By using raw scores and by choosing variables that have moderate diagnostic influence, e.g., choosing total learning over delayed recall from RAVLT, and only two variables out of >100 used in the memory clinic examination, we could remove most but not all of this handicap. Furthermore, many elderly have impaired vision and this may bias any visual task. In the present study, identifying a clearly visible “X” at arms lengths distance should not be seriously distorted by even moderate sight impairment. Another possible limitation is that the age-adjusted IN scores indicated group differences with respect to premorbid FSIQ. The relatively high and similar MMSE-SR scores, however, support that the sample consisted of the very intermediate cases of SCI, MCI, and mild-to-moderate dementia. A more practical limitation is that the methods to gather these variables are not fully automated. More work is needed regarding prediction automation, maybe similar to how we provide the actual models as an online decision support system with the present paper.

Improved medical care and general living conditions renders an aging population. An increasing number of individuals live long enough to develop MCI and dementia. As these numbers increase, so does the burden on individuals, families, organizations, and societies. Importantly, we found that different variables extracted from a 5 min SRT task could differentiate both MCI/dementia and SCI/MCI/dementia together with established tests. This is the primary clinical purpose of the established tests, as they are presently applied worldwide in costly and time-consuming neuropsychological assessments. Although in need of more research, the present findings suggest that SRT variables can contribute to diagnostic screening of these patients in conjunction with a few other established tests. The result from such screenings could then guide the tailoring of the main neuropsychological batteries for these patients. Insofar as several of the established tests demand the clinician's full attention during 5–15 min of testing, while SRT administration and scoring can be almost fully automated, SRT is particularly cost-effective.

## Conclusions

Cost-effective SRT variables, in conjunction with other established psychometric variables, hold potential for differentiating MCI/dementia, and SCI/MCI/dementia in the difficult-to-differentiate patients referred for full neuropsychological assessment as part of the memory clinic examination. Implementation in diagnostic support systems based on machine learning holds promise. External validation is needed.

## Declarations

### Ethics approval and consent to participate

The study was approved by the Regional Ethics Committee in Stockholm (Dnr: 2015/1493-31/1). Written consent was obtained by the neuropsychologist from capable patients, and in those cases where dementia compromised capacity then assent from the patient and written consent from a relative, according to local law and process, was obtained. If consent was not given, with or without reason, the index test was not administered. The patient was excluded from the study but continued the memory clinic investigation as planned. This procedure also applied if patients aborted testing or later withdrew participation. All aspects of the study adhere to the Declaration of Helsinki.

### Consent for publication

The manuscript does not contain any distinguishable individual data for which further consent is required.

### Availability of data and material

The datasets generated and/or analyzed during the current study are not publicly available. According to Swedish law, the individual clinical data is classified as sensitive personal information, and is therefore not freely available. We have no right to redistribute this data, as the informed consent signed by the patients in the present study explicitly states that no one except the researchers involved in the present research project are allowed to access data. Data are however available from the authors upon reasonable request and with permission by the Ethics Committee, involving an independent application to the Ethics Committee according to Swedish law.

## Author contributions

JW, EW, MA, BT, JU, and UE designed the study, collected data, interpreted the findings, critically revised the manuscript, designed the application, and approved its final form and submission. JW analyzed data and drafted the manuscript.

### Conflict of interest statement

The authors declare that the research was conducted in the absence of any commercial or financial relationships that could be construed as a potential conflict of interest.

## Footnotes

^1^*ICD-10 Classifications of Mental and Behavioral Disorders*. Clinical Descriptions and Diagnostic Guidelines. Available online at: http://apps.who.int/classifications/icd10/browse/2010/en

^2^*The Memory Clinic Diagnostic Prediction Instrument (MC-DPI)*. Available online at: http://wallert.se/mcdpi.
