# Supplementary material for: Differentiating Patients at the Memory Clinic With Simple Reaction Time Variables: A Predictive Modeling Approach Using Support Vector Machines and Bayesian Optimization
Source: Front Aging Neurosci. 2018 May 22;10:144. doi: 10.3389/fnagi.2018.00144 (PMC5972201; doi:10.3389/fnagi.2018.00144)
Supplement: Supplementary file 1 [file Data_Sheet_1.docx]

Appendix

Non-significant rank-sum tests over all three groups in Table 3 for SRT-µ (rank sum χ^2^ = 4.48, P = .107) and SRT-WP5 (rank sum χ^2^ = 4.86, P = .088) were not followed up with post-hoc testing. Significant first tests for SRTS-mean (rank sum χ^2^ = 12.71, P < .001), SRTS-sd (16.77, P < .001), SRTS-median (9.37, P = .009), SRT-mean (16.61, P < .001), SRT-sd (19.82, P < .001), SRT-median (14.16, P < .001), SRT-σ (9.95, P = .007), SRT-τ (15.75, P < .001), IN FSIQ (34.74, P < .001), MMSE (29.94, P < .001), BD (27.75, P < .001), DS (24.67, P < .001), RAVLT (31.94, P < .001), BNT-60 (14.72, P < .001), WMS (30.95, P < .001), RCFT (35.29, P < .001), TMT4 (45.91, P < .001), VFT3 (33.90, P < .001), Clock test (44.96, P < .001), Draw Cube (25.20, P < .001), and Draw Cross (19.84, P < .001) were followed-up.

Post-hoc tests for SRT variables in Table 3 show that SRTS-mean was significantly faster for SCI vs MCI (Dunn test = -2.64, P < .001) and for SCI vs dementia (-3.56, P < .001) but not for MCI vs dementia (-1.33, P = .092), SRTS-sd was significantly less variable for SCI vs MCI (-3.25, P = .001) and for SCI vs dementia (-4.09, P < .001) but not for MCI vs dementia (-1.22, P = .112), SRTS-median was significantly faster for SCI vs MCI (-1.68, P = .047) and for SCI vs dementia (-2.95, P = .005), but not for MCI vs dementia (-1.83, P = .067), SRT-mean was faster for SCI vs MCI (-2.43, P = .015) and for SCI vs dementia (-3.98, P < .001) and for MCI vs dementia (-2.24, P = .013), SRT-sd was significantly less variable for SCI vs MCI (-3.58, P < .001) for SCI vs dementia (-4.44, P < .001) but not for MCI vs dementia (-1.26, P = .105), SRT-median was not significantly faster for SCI vs MCI (-1.75, P = .039) but significantly faster for SCI vs dementia (-3.51, P < .001) and for MCI vs dementia (-2.53, P = .012), SRT-σ was significantly less variable for SCI vs MCI (-2.35, P = .019) and for SCI vs dementia (-3.15, P = .002) but not for MCI vs dementia (-1.17, P = .122), SRT-τ was significantly faster for SCI vs MCI (-3.48, P < .001) and for SCI vs dementia (-3.87, P < .001) but not for MCI vs dementia (-0.59, P = .278), SRT-WP5 was significantly faster for SCI vs MCI (-3.06, P = .002) SCI vs dementia (-4.62, P < .001) and MCI vs dementia (-2.26, P = .012).

Corresponding follow-up testing was conducted for the established psychometric tests in Table 3, showing that IN FSIQ was significantly higher for SCI vs MCI (Dunn test = 3.42, P < .001) SCI vs dementia (5.74, P < .001) and MCI vs dementia (3.34, P < .001), MMSE score was higher in SCI vs MCI (2.51, P = .006) SCI vs dementia (5.11, P < .001) and MCI vs dementia (3.65, P < .001), BD score was higher for SCI vs MCI (3.09, P = .002) SCI vs dementia (5.14, P < .001) and MCI vs dementia (2.95, P = .002), DS score was higher for SCI vs MCI (2.00, P = .023) SCI vs dementia (4.50, P < .001) and MCI vs dementia (3.58, P < .001), BNT-60 score was higher for SCI vs MCI (2.29, P = .022) SCI vs dementia (3.73, P < .001) and MCI vs dementia (2.15, P = .016), RAVLT score was higher in SCI vs MCI (3.73, P < .001) SCI vs dementia (5.60, P < .001) and MCI vs dementia (2.70, P = .003), WMS score was higher for SCI vs MCI (3.18, P = .002) SCI vs dementia (5.45, P < .001) and MCI vs dementia (3.04, P = .001), RCFT score was higher in SCI vs MCI (3.59, P < .001) SCI vs dementia (5.89, P < .001) and MCI vs dementia (3.41, P < .001), TMT4 was faster completed in SCI vs MCI (-3.10, P < .001) SCI vs dementia (-6.33, P < .001), and MCI vs dementia (-4.66, P < .001), VFT3-shifting score was higher in SCI vs MCI (2.75, P = .003) SCI vs dementia (5.46, P < .001) and MCI vs dementia (3.88, P < .001), Clock Test score was higher in SCI vs MCI (2.45, P = .007) SCI vs dementia (5.92, P < .001) and MCI vs dementia (5.06, P < .001), Draw Cube score was not significantly higher in SCI vs MCI (1.44, P = .075) yet was significantly higher for SCI vs dementia (4.22, P < .001) and MCI vs dementia (4.05, P < .001), Draw Cross score was not significantly higher in SCI vs MCI (1.47, P = .071) but was significantly higher for SCI vs dementia (3.84, P < .001) and for MCI vs dementia (3.48, P < .001) groups.
